# Blood oxygen level–dependent functional magnetic resonance imaging can evaluate the efficiency of transcatheter arterial chemoembolization in hepatocellular carcinoma

**DOI:** 10.1016/j.jimed.2019.05.002

**Published:** 2019-06-27

**Authors:** Lizhi Xiao, Enhua Xiao

**Affiliations:** a The Center of PET-CT, The Second Xiangya Hospital of Central South University, Changsha, 410011, Hunan, China; b Department of Radiology, The Second Xiangya Hospital of Central South University, Changsha, 410011, China

**Keywords:** Blood oxygen level–dependent, Magnetic resonance imaging, Efficiency, Hepatocellular carcinoma, Transcatheter arterial chemoembolization

## Abstract

Hepatocellular carcinoma (HCC) is among the most common malignant tumors worldwide, and transcatheter arterial chemoembolization (TACE) technology has become the first-line treatment for advanced HCC. Another important, recently developed technique is blood oxygen level–dependent functional magnetic resonance imaging (BOLD-fMRI), which utilizes hemoglobin as an endogenous contrast agent and measures deoxygenated hemoglobin content by sampling the oxygen content of tissues, thus reflecting the hemodynamics and pathophysiologic changes in body organs. Currently this technology is being used in patients with liver tumors; that is, it serves as an important tool in follow-up after TACE. The present paper summarizes these developments.

## 1 Introduction

Hepatocellular carcinoma (HCC) is among the most common malignant tumors, causing more than 600,000 deaths annually.^1^^,^^2^ In terms of fatalities due to cancer,^1^ it is exceeded only by pulmonary carcinoma and gastric carcinoma.

There are several effective treatment approaches for early-stage HCC, including surgical excision, transplantation, and radiofrequency ablation. Nevertheless, in more than 70% HCC patients, the disease is detected only at a middle or advanced stage, making many of these treatment approaches unsuitable.^3^, ^4^, ^5^ Fortunately, transcatheter arterial chemoembolization (TACE) can effectively treat middle- or advanced-stage HCC by embolizing the arteries surrounding the tumor and injecting chemotherapeutic drugs into the tumor. Because of its superiority to other methods, TACE has been recommended as the first-line therapeutic method for middle-to advanced-stage HCC.^5^

Imaging examinations play an increasingly significant role in the follow-up after TACE in patients with HCC. Another important, recently developed technique is blood oxygen level–dependent functional magnetic resonance imaging (BOLD-fMRI), which utilizes hemoglobin as an endogenous contrast agent and measures deoxygenated hemoglobin content by sampling the oxygen content of tissues, thus reflecting the hemodynamics and pathophysiologic changes in body organs. At present, this method has been used in the detection of lesions in multiple organs and organ systems, including liver tumors. It can therefore serve as an important follow-up detection technique in HCC patients who have undergone TACE.^6^

## 2 TACE for the primary treatment of HCC

TACE uses a percutaneous interventional technique to cut off the liver tumor's blood supply; it does so by embolizing branches of the hepatic artery and then injecting chemotherapeutic drugs into the tumor, thus resulting in the apoptosis and necrosis of the malignant cells.^7^ The results of previous meta-analyses show that TACE can significantly extend the survival of patients with HCC.^8^^,^^9^

Although TACE is effective in inducing the apoptosis and necrosis of HCC cells, some studies suggest that TACE may also promote angiogenesis by inducing hypoxia, facilitating proliferation of residual HCC cells, and promoting the activation of angiogenesis-related factors, such as vascular endothelial growth factor (VEGF).^10^ Other studies indicate that the high concentration of insulin-like growth factor-2 (IGF-2) in HCC patients may serve as an independent risk factor of post-TACE metastasis of the tumor.^11^ Therefore, it is extremely important to determine the precise therapeutic effects of TACE.

Several factors can account for the recurrence of post-TACE HCC, including changes in the blood supply of the tumor, such as a portal vein supply to the tumor's marginal sections; failure of TACE to fully embolize or recanalize the tumor's supply vessels; the formation of a collateral blood supply; opening of the tumor's potential communicating branches; angiogenesis of the tumor; and partial hepatic arteriovenous fistula.^12^ Additional variables include the numbers, sizes, and differentiation levels of the primary tumor; degree of liver function and of hepatic cirrhosis; and possible liver damage during or after TACE. All of these may contribute to the recurrence of HCC. The early detection of residual and/or recurrent tumor can facilitate early treatment and help to improve the survival rate.

## 3 Post-TACE detection methods by imaging

Imaging detection methods play an increasingly significant role in the follow-up of HCC patients after TACE. Currently used imaging modalities include Doppler color-flow imaging (DCFI), digital subtraction angiography (DSA), computed tomography (CT), and magnetic resonance imaging (MRI). Each of these has advantages and disadvantages. DCFI is sensitive to early diagnosis while it also results in high-false positive rates. DSA is effective in evaluating the therapeutic effect of TACE, yet its invasiveness impedes its application as a routine examination. Dynamic enhanced CT can clearly show the status of residual tumor necrosis and new lesions, but highly iodinated contrast agents can also enhance the cancer survival zone. Conventional MRI can sensitively detect new cancer lesions after surgery.^15^ Furthermore, there is a common deficiency of the aforementioned methods—namely, they cannot determine the oxygen content of HCC tissues or show its changes.

## 4 BOLD-fMRI in post-TACE HCC

### 4.1 Fundamental principles of BOLD-fMRI

In the 1930s, Pauling et al.^16^ pointed out that deoxyhemoglobin is a paramagnetic substance; the hemoglobin and deoxyhemoglobin in the blood have opposite magnetic properties, and the magnetism of the blood is dependent on the hemoglobin's level of oxygenation. Thereafter, Ogawa et al.^17^ proposed that the paramagnetic deoxyhemoglobin in blood could serve as a natural contrast agent in MRI scanning. That is, researchers could measure the oxygen content of microvessels by using a gradient echo pulse sequence in a high magnetic field.

Paramagnetic deoxyhemoglobin can create a magnetic field gradient and cause asymmetry in local tissues within the magnetic field by forming a smaller magnetic field, thus shortening the T2-weighted signal. When the blood supply increases, the hemoglobin will increase and the paramagnetic deoxyhemoglobin's effect in shortening the T2 signal will be weakened, thus lengthening the T2 signal of corresponding tissues. The opposite effect will occur when the deoxyhemoglobin increases, thus shortening the T2 signal.^18^ BOLD-fMRI is based on these principles. It depends on changes in the oxygen content of normal and abnormal tissues based on the hemodynamics of the tissues and/or organs being studied. As the transverse relaxation rate (Rf) is directly relevant to the concentration of deoxyhemoglobin in tissues, Rf (R2* ​= ​l/T2*, unit Hz) is often adopted as an indicator in the assessment of changes in oxygen content.^19^ BOLD-fMRI uses a 1.5T or 3.0T MRI system to scan with a BOLD T2* sequence or transverse section R2* sequence. In image processing, researchers can then calculate the R2* or T2* value by measuring the mean value of the region of interest (ROI) using specific measuring tools.^19^, ^20^, ^21^, ^22^, ^23^, ^24^

### 4.2 Clinical application of BOLD-fMRI

BOLD has been widely used in studying the central nervous system (CNS).^25^ There has also been progress in applying BOLD in studying diseases of the kidney^26^ and prostate.^27^ The application of BOLD in HCC is in its early stages, and there are not as yet any large-scale clinical trials in this field.

Haque et al.^20^ hypothesized that BOLD-fMRI could show changes in oxygen consumption in pig liver as well as in the human body by using glucose as stimulant. Yang et al.^21^ studied normal rabbits as well as a rabbit liver fibrosis model by using BOLD-fMRI scanning; they found that the Rf value increased as the pathologic stage of fibrosis progressed, but BOLD-fMRI was not useful for staging early fibrosis, nor could it stage or grade fibrosis explicitly. Nevertheless, BOLD-fMRI is effective in diagnosing severe liver fibrosis and cirrhosis; it can also reflect the severity of liver fibrosis to some extent, thus providing a new way of guiding clinical treatment.

The use of BOLD-fMRI to evaluate the oxygen content of liver tumors and facilitate differential diagnosis has good prospects for clinical application. Rhee et al.^22^ used polyethylene alcohol particles to embolize rabbit VX2 tumors by TACE and then used BOLD-fMRI to evaluate the therapeutic effect. They found a significant decrease in the T2* value after TACE, which is congruent with a decrease in the blood oxygen saturation of liver tumors and reflects a decrease in abnormal blood supply to tumor tissues. This indicates that the use of BOLD to detect changes in the blood oxygen content of liver tumors after embolization is feasible. Yu et al.^23^ applied pretreatment BOLD detection to 35 patients with benign liver tumors, 62 patients with malignant liver tumors, and 12 patients with liver abscesses. The results showed that the Tf value, lesion/muscle ratio, and lesion/spleen ratio in malignant liver tumors were all significantly lower than those in benign liver lesions, suggesting that T2*-relevant values were useful in differentiating benign versus malignant liver tumors and that the Tf value and its lesion/muscle ratio can be used as a parameter in the diagnosis of liver abscess. In 16 patients with primary HCC, Luo et al.^19^ used BOLD-fMRI to detect oxygen before and after treatment with high-intensity focused ultrasound. Two weeks after treatment, they found a significant increase in the Rf value and a significant decrease in the T2* value compared with pretreatment, implying that BOLD-fMRI may be useful in evaluating oxygen uptake in primary HCC before and after treatment with high-intensity focused ultrasound. Dai et al.^24^ compared the pre- and post-TACE BOLD-fMRI scans of 10 primary HCC patients and further compared the BOLD scans of the 10 patients with those of 10 healthy controls; they found no statistically significant differences between the HCC patients and healthy controls in T2* values of liver parenchyma, whereas primary HCC patients showed a significant decrease in T2* values of the tumor tissues with rich blood supplies and a significant increase in the T2* values of the liver tissues surrounding the tumors. These findings showed that using BOLD-fMRI technique to evaluate the pre- and post-TACE changes of oxygen content in both the tumor and normal tissues of HCC patients is both feasible and practical. The BOLD-fMRI technique has huge potential and can be of particular value in following the progression and treatment of liver tumors. However, it still requires further study via large-scale clinical trials.

### 4.3 Disadvantages of BOLD-fMRI

In the analysis of BOLD-fMRI scanning data, the R2* value is measured in a manually selected region of interest, which is easily affected by partial volume effect, the distribution of blood vessels, necrosis and bleeding, the radiofrequency pulse parameter, and subjective factors.^21^ This technique is also easily affected by plasma proteins, molecular diffusion, pH value, temperature, pixel size, and the direction of blood and vascular flow. Furthermore, because iron, stored in the liver, is a paramagnetic substance, it may also affect the results of BOLD-fMRI.

## 5 Summary

TACE is now the first-line treatment for middle and advanced liver cancer; however, further studies evaluating its efficacy are needed. BOLD-fMRI as a burgeoning noninvasive method for evaluating hypoxia in tumors still has disadvantages. With further development, BOLD-fMRI may find a place in clinical practice owing to its noninvasiveness and high repeatability, and its application in evaluating the efficacy of TACE will also be improved increasingly.
